# Honey Robbing: Causes, Impacts and Preventive Measures

**DOI:** 10.3390/insects16010015

**Published:** 2024-12-27

**Authors:** Xinyu Wang, Ting Huang, Quanzhi Ji, Jun Guo, Yazhou Zhao

**Affiliations:** 1 Institute of Apicultural Research, Chinese Academy of Agricultural Sciences, Beijing 100093, China; 82101235491@caas.cn (X.W.); 18083445691@163.com (T.H.); 18336369851@163.com (Q.J.); 2 Faculty of Life Science and Technology, Kunming University of Science and Technology, Kunming 650500, China

**Keywords:** honey bee, honey robbing, behavioral characteristics, causes, hazards, occurrence mechanism

## Abstract

Honey robbing, which typically occurs during times of food scarcity, is a perilous foraging strategy for bee colonies and presents a formidable challenge in the realm of beekeeping. This article provides a comprehensive and multifaceted exploration of honey robbing, including the morphology, behavioral traits, timing, and scope of this phenomenon. This exploration elucidates the specific manifestations of honey robbing, offering readers a deeper understanding of its various facets. Next, this article investigates the root causes of honey robbing by examining both abiotic and biotic factors. The resulting harms are outlined, and corresponding preventive and control measures are suggested. Finally, the article succinctly summarizes the current obstacles in research related to honey robbing and outlines promising avenues for future exploration. The objective of this study was to elucidate the occurrence mechanism of honey robbing, ultimately aiming to contribute to the sustainable growth of the beekeeping industry.

## Simple Summary

Honey robbing refers to a situation where, during times of food scarcity, worker bees are forced to adopt more aggressive and risky foraging strategies to ensure the survival and reproduction of their own colony. This involves invading other colonies to pillage their food resources. Such behavior poses a serious threat to both bee populations and beekeepers. This review focuses on the morphological and behavioral characteristics of honey robbers and elaborates on the timing, distribution, and causes of honey robbing. The article outlines the various harms caused by honey robbing and proposes effective prevention and control measures accordingly. Furthermore, we summarize the potential obstacles currently facing honey robbing research and lay out feasible paths for future exploration. The aim of this paper is to assist beekeepers in managing their colonies more effectively and thereby promote the sustainable development of the beekeeping industry.

## 1. Introduction

In autumn in the Northern Hemisphere, as natural sources of nectar and pollen gradually dwindle, it is common to see bees engaging in disorganized, erratic circular flights around their colonies [1,2,3]. The term “honey robbing” refers to a phenomenon that arises in nature when there is a severe reduction in honey flow, or when nectariferous plants are coming to an end and nectar production is significantly reduced. To ensure the survival and reproduction of their colony (the robbing colony), worker bees are forced to adopt a more aggressive and risky foraging strategy, which involves invading another bee colony (the robbed colony) to seize its food resources [1,2,4]. When a colony is vulnerable due to a weak defensive system or poor management, its stored food can easily fall prey to raiding colonies, thereby exacerbating the problem of honey robbing [1]. This phenomenon is common in colony management, and similar behavioral patterns have been observed in other social bee species, such as *Apis florea* [5], *A. dorsata* [6], and the stingless bee (*Trigona*) [7], emphasizing the widespread occurrence of this behavior. Honey robbing has long been a highly respected and intricate area of focus within the field of insect sociobehavioral science. Research into honey robbing has significantly improved our understanding of insect social structures and resource-allocation systems while also demonstrating the remarkable adaptability and strategic prowess of insects when facing environmental challenges. Currently, research on honey robbing covers a broad spectrum, including behavioral traits, triggering mechanisms, transmission pathways, impact patterns, and potential preventive and control strategies. The relevant research findings provide novel insights and strategies for the effective protection and breeding of social insects, especially honey bees.

## 2. Characteristics of Honey Robbing

### 2.1. Identification of Honey Robbing

Before intensive robbing occurs, when intense battles are evident throughout an entire colony, beekeepers typically observe at the hive entrance only attempts of robbery, prying, prowling, crawling, or raids by foreign workers during late summer [1,8]. Although such a colony is capable of defending itself, robber bees merely attempt to infiltrate the colony. Conversely, during honey robbing, if the colony being targeted lacks the ability to resist, it becomes helpless, and its honey is entirely plundered by the robber bees [1,8]. The robber bees in the robbing colony play a leading role in foraging activities. These bees swiftly and precisely adjust their foraging strategies, seizing the chance to infiltrate the unprotected colony under attack [1,8]. Once inside, the bees collect honey from the honeycombs, carry it back to their colony, and recruit more bees to join the “theft”.

**Figure 1:**
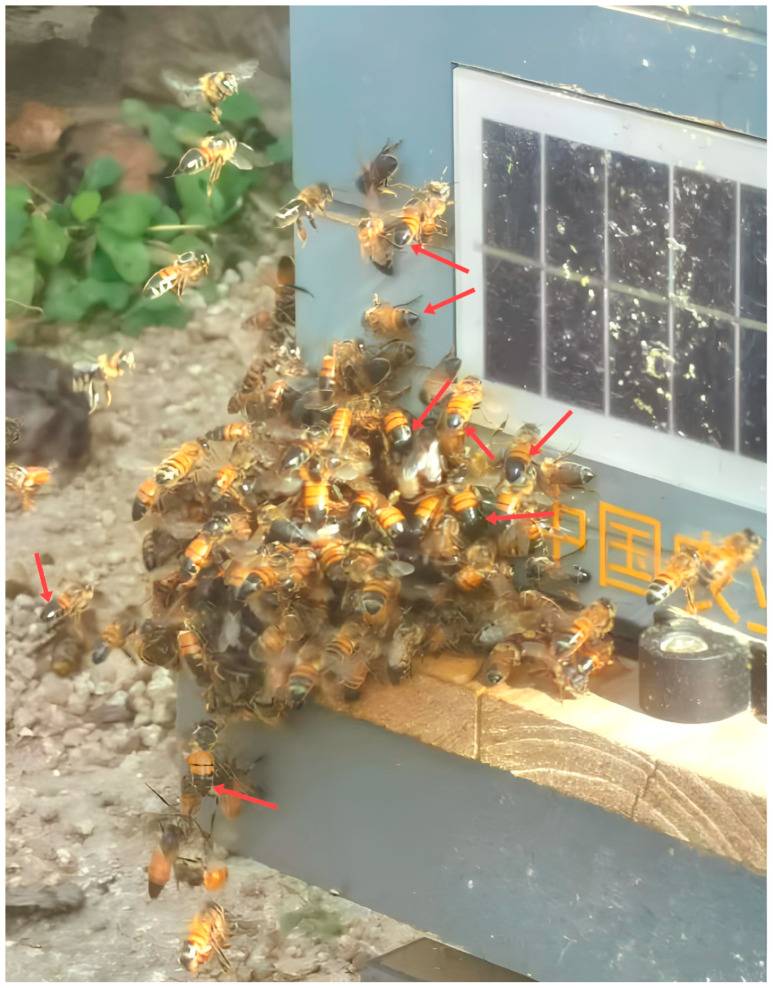
The robber bees gathering at the entrance to a hive (red arrows point to robber bees).

Robber bees display distinctive morphological traits, including shedding most of the hair from their bodies, revealing dark, glossy abdomens that may hint at their advanced age or the numerous fierce battles they have endured (Figure 1) [1,8,9]. In addition to their morphological characteristics, the behavioral patterns of robber bees are equally pivotal in their identification [1,8,10,11]. Around the colony that has been robbed, chaotic scenes often unfold, with numerous worker bees flying in disarray and their wings vibrating sharply as if in a frenzy. These robber bees tend to gather at the cracks and joints of the hive, and the ground is littered with numerous dead bees with curved abdomens and bitten combs [10,11,12]. The robber bees often enter the robbed colony on an empty stomach and emerge with a full stomach. In front of the nest entrance of the robbing colony, worker bees frequently enter and exit and are as busy as they are during the main honey-flowing period [1,12]. Close observation can show that the worker bees entering the colony have full abdomens, whereas those exiting have relatively smaller ones. In addition, the bees from these robbing colonies start foraging earlier and end later than those from normal colonies do [1,9]. It is possible to verify a robbing colony by sprinkling white flour near the colony entrances and observing the bees entering each colony. Once bees covered with white powder are found flying into a specific colony, it can be concluded that this colony is the robbing colony [8,13].

### 2.2. Time and Scope of Occurrence of Honey Robbing

In the Northern Hemisphere, honey robbing can occur throughout the year, but it is most common and severe during late autumn [1,14]. This timing is because bees naturally want to stockpile enough food for the winter. Even if there may still be sporadic nectar sources outside at this time, the low temperatures make it difficult for bees to forage, which can quickly lead to honey robbing. Meanwhile, in northern China, lower temperatures in early spring may lead to delayed blooming of nectariferous plants, which in turn may result in instances of honey robbing. Additionally, honey robbing can also occur if there is a sustained period of high temperatures and sunny weather [14,15,16]. This phenomenon occurs because bee colonies striving to fulfill the requirements for brood rearing or temperature maintenance become highly vulnerable to the stress of food scarcity [17], ultimately resulting in the act of stealing honey from other colonies.

Honey robbing is widespread among various species of bees and is not limited solely to interactions among bees of the same species; honey robbing can also occur between colonies of different bee species [5,6,7]. The bee species that are most commonly domesticated worldwide are *A. cerana* and *A. mellifera*. Owing to their relatively small size, quick response speed, and extreme sensitivity to odors [18,19], *A. cerana* bees tend to exhibit a greater incidence of honey robbing behavior than *A. mellifera* bees do. However, when *A. cerana* bees engage in honey robbing with larger *A. mellifera* bees, *A. cerana* colonies often find it difficult to resist the counterattacks of *A. mellifera* bees, ultimately leading to potential colony collapse. Honey robbing typically occurs first between adjacent colonies within the same apiary but can spread to different apiaries in severe cases. Therefore, to avoid these conflicts, *A. cerana* apiaries and *A. mellifera* apiaries should be separated as much as possible to reduce the occurrence of honey robbing.

## 3. Causes of Honey Robbing

### 3.1. Environmental Factors

Environmental factors constitute one of the root causes of honey robbing and can be divided into two categories: the apiary environment and the natural environment.

When an apiary is located in a small basin environment enclosed by high walls, hills, or accumulated debris, this terrain can result in inadequate natural ventilation conditions at the apiary. In this environment, the air within the apiary is often thick with the scent of wax, propolis, and honey [19]. These persistent aromas can easily trigger the sensitivity of scout bees, especially during periods of high temperatures or intense sunlight, providing favorable conditions for honey robbing. In addition, the quality of the water sources surrounding the apiary plays a crucial role. Nearby water sources contaminated with antibiotics or pesticides can impair the olfactory ability of bees [20,21], reducing the colony’s defense against robbing and consequently increasing its vulnerability to attacks by robber bees. The type of nectariferous plants surrounding an apiary also influences the frequency of honey robbing, potentially due to differences in nectar composition, volatile components, and sugar concentration among plants [22,23]. Research has shown that nectar with stronger odors and higher sugar concentrations attracts more honey bee [19,23].

In addition to the layout and management of the apiary, natural environmental factors such as temperature and humidity significantly affect the occurrence of honey robbing [24]. Under prolonged cold or hot and dry weather conditions, the flight activities of bees are suppressed, affecting the normal supply of food resources to the colony [15,16,17]. This disruption can make the colony irritable, thereby increasing the risk of honey robbing. Furthermore, climate change is gradually disrupting the natural growth balance of plants, affecting the harmonious relationship between the flowering period of nectariferous plants and bees’ foraging activities [25,26]. First, when the rhythm of the natural climate is disrupted by external factors, bees find it difficult to locate sufficient food reserves. In the face of food shortages, also called a “food crisis”, bee colonies may adopt more aggressive foraging strategies, such as robbing food resources from neighboring colonies, to sustain their survival, which leads to honey robbing [1]. Second, the widespread monoculture model of modern agriculture, while providing bees with an adequate source of nectar and pollen in the short term, results in a rapid depletion of honey sources once the flowering season concludes [27]. This sudden interruption of nectar sources often leads to a greater incidence of honey robbing among colonies. Third, the period of seasonal transition is a critical time that affects the food supply for colonies [28,29]. In particular, the swift shift from the bounty of nectar sources in summer to the scarcity of food in autumn often serves as a pivotal trigger for honey robbing. Therefore, it is crucial for beekeepers to understand and adapt to these changes in the natural environment and to implement suitable management measures to effectively prevent and control honey robbing.

### 3.2. Colony Management

The professional skills and management strategies of beekeepers are also key factors that influence honey robbing [1,30]. Excessive and frequent opening of hives by beekeepers for inspection not only disrupts the normal order of the colony, causing bees to be in a disturbed and chaotic state but may also trigger honey robbing because frequent inspections inadvertently release the honey aroma within the hive, attracting bees from nearby colonies to probe or even steal from the hive [30]. During periods of nectar and pollen scarcity in nature, the failure of beekeepers to perform artificial feeding in a timely and appropriate manner [31,32]—for example, delaying feeding leading to hunger in the colony or providing sugar water with improper concentrations or pollen of poor quality—will result in food shortages within the colony, thereby inducing honey robbing. The management of hive entrances is equally important. Failure to adjust the size of hive entrances in a timely manner according to seasonal changes and weather conditions can also induce honey robbing [30]. A hive entrance that is overly restricted can impair air circulation within the hive, enabling the honey scent to leak through any gaps in the hive and attract robber bees. On the other hand, an overly large hive entrance may make it more convenient for robber bees to easily invade the colony. If beekeepers are not punctual in their daily inspection and maintenance work on the colonies or fail to promptly identify and address issues such as queenless colonies and weak or sick colonies, opportunities for robber bees will increase, as will the risk of honey robbing [30,33].

### 3.3. Biological Factors

Honey robbing, a unique and complex behavioral pattern within bee populations, has its roots deeply embedded in various biological factors. Among them, the genetic differences among colonies are important factors contributing to variations in aggressiveness and predatory behavior exhibited by foraging bees. A notable example is the Africanized bee (*A. m. scutellata*), which is extremely aggressive compared with the European bee (*A. m. mellifera*) [34]. Genetic differences not only shape the unique defense mechanisms of colonies but also profoundly influence their foraging strategies [35]. Therefore, when environmental pressures increase, such as during times of scarce nectar sources, these bee subspecies are more prone to honey robbing than other subspecies that are genetically more docile are. To ensure their own survival and reproduction, aggressive subspecies will not hesitate to steal food from neighbouring colonies. Furthermore, a high royal jelly-producing strain of bees in China known for its heavy brood-rearing tasks, large colony sizes, and immense food demands make them more susceptible to honey robbing than other varieties are [36]. From this point of view, tackling the survival challenges arising from this inheritance cannot be addressed solely through beekeeping practices. Genetics and biotechnology can be used to understand the specific mechanisms involved and to reduce the incidence of honey robbing to a minimum in a safe and secure manner.

The collection and storage of nectar are essential survival skills for bees that are deeply ingrained in their natural behavior. In extreme cases, bees engage in honey robbing, redistributing food resources by stealing honey from other colonies [37]. To a certain extent, this process reflects internal competition within natural bee populations [38,39], effectively promoting the survival of strong colonies while naturally eliminating those that are weaker, diseased, or have lost their queen bees. Therefore, honey robbing is not only a strategy for bee populations to cope with resource scarcity but also an inherent aspect of intraspecies competition and optimization. From an evolutionary perspective, over the long term, honey robbing may represent a complex adaptive strategy in response to fluctuations in environmental resources, potentially intertwined with various evolutionary processes. Yet, in the short term, such behavior hinders the stable development of colonies and somewhat diverges from the typical evolutionary trajectory that promotes population sustainability.

## 4. Impact of Honey Robbing on Beekeeping

### 4.1. Impact on the Robbed Colony

Honey robbing has a direct effect on the productivity of the robbed colony. The aggressive behavior of robber bees accelerates the draining of the colony’s food supplies, especially when honey flow is scarce, making it harder for the robbed colony to survive and severely weakening their ability to forage and defend themselves. More seriously, the intense fighting between the foraging bees on both sides triggered by honey robbing often results in significant casualties, causing a sharp decline in the colony strength of both colonies [1,14]. Honey robbing constitutes a social ill among honey bees. In extreme cases, a colony that has been robbed can find its honey reserves completely depleted, with larvae and pupae being dragged out and harmed. In some instances, the queen bee may even be injured or killed [1,10,11], ultimately compelling the entire colony to abandon their hive and flee [40]. This cascade of events not only seriously undermines the stability of the robbed colony but also impairs the long-term viability of the robbing colony. Furthermore, honey robbing increases the level of interaction between the robbed and robbing colonies, creating greater opportunities for the spread of pests and diseases [41], which in turn jeopardizes the health of the bees in the robbed colony.

### 4.2. Impact on the Robbing Colony

Honey robbing is a survival strategy employed by the robbing colony, ensuring its own continuation by plundering food resources from other colonies. However, while this intense resource-plundering behavior may meet the survival needs of the robbing colony in the short term, it also carries enormous costs. Research has shown that this predatory behavior significantly depletes the energy reserves of robber bees and may even shorten their natural lifespan [41]. As highly social insects, the stability of the social structure of bees relies on the strict division of labor and cooperation [42]. To successfully execute a raid, the robbing colony needs to dispatch many foraging bees to perform tasks such as reconnaissance, raiding, and transporting resources. These foraging bees need to not only navigate precisely and engage in intense combat in complex external environments but also communicate and coordinate effectively with their fellow bees [43]. However, these large-scale operations often disrupt the internal division of labor within the robbing colony [44,45], undermining its overall organizational efficiency and social structure. This disruption, in turn, weakens the colony’s ability to collect nectar and protect itself, sometimes even making it a target of predation by other colonies. Furthermore, studies have indicated that robber bees are more susceptible to infection by pathogens such as *Varroa* mites and *Nosema* spores [46,47]. This finding demonstrates that the frequent interactions between the robbing colonies and the robbed colonies not only endanger the health of the bees in the robbed colonies but also increase the risk of disease and parasite infection in the robbing colonies. Thus, while honey robbing may offer immediate survival benefits to the robbing colonies, profound negative consequences for their population’s health and overall survival prospects can ultimately occur.

### 4.3. Impact on the Beekeeping Industry

Honey robbing may initially be confined to individual colonies, but if prompt measures are not taken to control it, its impact can quickly spread, affecting the entire apiary [8,10], similar to a “behavioral contagion”. When honey robbing becomes frequent or widespread, the bees’ alertness and aggressiveness increase significantly, rendering them unusually fierce. This change undoubtedly complicates colony inspections and feeding, severely disrupts normal breeding and production routines, and presents a significant challenge to beekeepers’ management efforts. For example, in Himachal Pradesh, India, in 2001 and 2002, the loss of bee colonies due to honey robbing ranged from 15% to 75%, with a loss of colony strength of approximately 30.10% and a decrease in brood-rearing efficiency of 42.49% [48]. Meanwhile, Seiler et al. found honey robbing increased risks of contamination of honey in the robber colony with antibiotics or other contaminants, and when robbing occurs it is also more difficult to control the quality of honey [49]. To combat honey robbing, beekeepers must dedicate significant time, energy, and financial resources to enacting a range of management strategies, all aimed at halting the spread of honey robbing and the damage caused by this scenario [1]. However, the adoption of these measures not only complicates the management process but also substantially elevates beekeeping costs, thereby placing an extra financial strain on beekeepers. Over time, this strain may adversely affect beekeepers’ overall income and jeopardize the sustainable growth of the beekeeping industry.

## 5. Strategies to Prevent and Manage Honey Robbing

### 5.1. Improving Colony-Management Practices

Honey robbing primarily occurs when bees use their sense of smell to find and steal honey from other colonies. Hence, a crucial aspect of preventing honey robbing is to prevent the honey scent from escaping the colony [1,8]. To achieve this objective, several preventative steps can be implemented. First, swiftly repairing any damage or cracks in the hive is crucial to guarantee optimal sealing. Second, ensuring that the air vents on the hive are not too close to the honeycombs prevents the sweet smell from escaping easily. Finally, trying not to open the hive too often to check on the bees can also help prevent the honey smell from escaping [30]. In addition, during the feeding process, attention should be paid to avoiding letting any honey or sugar water drip outside the hive, as this could attract robber bees. In the fall, it is advisable to feed bee colonies at night to minimize the risk of robber bees scouting and invading. It is important to remember that the combs, once honey has been extracted, should be returned promptly to all colonies, preferably no earlier than dusk and as comprehensively as possible. Moreover, it is crucial to quickly remove any discarded frames to avoid attracting robber bees. To increase the colony’s defense, the entrance to the hive can be narrowed to prevent robber bees from sneaking into the colony. Adding frames to the colony regularly ensures that ample space is available to survive and reproduce, which in turn increases the colony’s population and strengthens its resistance to external threats [50]. Site selection for the apiary is key to preventing honey robbing. Positioning the hives in an area with ample water sources and rich nectar can increase the bees’ foraging efficiency and the colony’s food reserves, significantly lowering the chances of honey robbing [51,52]. The proper arrangement of hives within the apiary is vital. Hives in the same row should be spaced 1–2 m apart, whereas those in adjacent rows should be 2–3 m apart and arranged in a staggered pattern [53]. This setup helps minimize inter-colony conflicts, reduces the incidence of honey robbing, and lowers the risk of disease transmission.

### 5.2. Strengthening Resource Security

An adequate supply of feed in a colony is essential to prevent honey robbing effectively [1]. As external nectar and pollen sources start to decrease, it is especially important to quickly provide colonies with capped honeycombs. If honeycombs are in short supply, feeding with high-concentration sugar syrup can serve as an effective alternative to mitigate the risk of honey robbing due to food scarcity [1,54]. Přidal et al. also reported the low attraction of the inverted sugar syrups for robber bees in comparison with sucrose syrups [55]. The best time to feed sugar syrup is in the evening, when bees are less active, preventing the confusion caused by competing for food during peak foraging times. The feeding amount should be adjusted so that the colony can finish it by dawn. This step ensures that the bees obtain enough food and prevents honey robbing induced by leftover syrup. Additionally, care should be taken during feeding to prevent any sugar syrup from dripping outside the hive. If the syrup accidentally spills, it should be rinsed off immediately with clean water to prevent the gathering of bees from other colonies and reduce the risk of honey robbing [30].

### 5.3. Adjusting the Structure of the Colony

Effectively managing the dynamic shifts in colony structure is crucial for minimizing the incidence of honey robbing. In beekeeping, a strong and healthy colony is essential, and balancing the social roles of worker bees within the colony is of utmost importance [56]. Ensuring that the bee population matches the number of combs or allowing the number of bees to slightly exceed the comb count can significantly decrease the chances of honey robbing. Timely replacement of the queen bee is a pivotal step in optimizing a colony’s structure and increasing its overall performance [31]. A new queen not only enhances the brood-rearing rate but also revitalizes a colony’s vitality and productivity, thereby strengthening its competitiveness and enabling it to more effectively address challenges and threats from the external environment [57]. Therefore, beekeepers must regularly evaluate the health of queen bees. If the queen is found to be in poor health or missing, prompt action should be taken to replace her with a new queen, ensuring the colony’s stable growth and ongoing reproduction. Beekeepers should adjust the size of their apiaries flexibly according to the actual needs of the colonies. Regularly splitting or merging colonies, as well as carefully managing the density of the apiary, is crucial for optimizing resource utilization. These practices not only enhance the resilience and adaptability of colonies but also significantly reduce the likelihood of unfavorable events, such as honey robbing. By implementing these strategies, beekeepers can ensure more efficient use of resources while bolstering the overall health and stability of their colonies [50].

### 5.4. Introducing Preventive Measures After Honey Robbing

Once honey robbing occurs, the apiary quickly descends into chaos, with bees flying in all directions, making hurried sounds, and gathering to fight around the robbed colony [1]. In this situation, water should be immediately sprayed into the air to calm the flying bees and prevent the honey robbing behavior from spreading further among the colonies [12]. Immediately afterward, smoke should be blown into the robbed colony to drive away any intruding robber bees. After the robbing colony is identified by sprinkling white flour at the entrance of the robbed colony, the locations of the robbing colony and the robbed colony can be swapped to confuse the robber bees and disrupt their original raiding routes [9,50]. Leveraging bees’ sensitivity to odors, irritating yet harmless scents can be emitted at the colony’s entrance by burning or spraying to discomfit the robber bees and encourage them to cease their predatory actions. Blocking is also an effective method to prevent honey robbing. Using branches or weeds to obscure the entrance of a robbed colony can hinder robber bees from finding and locating the colony. If the previous methods fail to halt honey robbing, drastic steps must be taken to stop its spread, including the complete elimination of the robbing colony. In extreme cases, if the entire apiary is affected by honey robbing, it may be necessary to relocate the entire apiary to a safe location more than 5 km away with abundant nectar sources [1].

## 6. Research Progress on Honey Robbing

### 6.1. Hotspots and Challenges in Honey Robbing Research

Honey robbing is an extreme foraging behavior adopted by bees when facing food scarcity, and the underlying regulatory mechanisms are complex. Currently, studies on honey robbing have been reported globally.

Researchers are committed to elucidating the decision-making mechanisms of colonies during honey robbing and have revealed that robber bees adapt their foraging strategies based on multiple factors, such as colony size and health, food quantity and quality, and accessibility, in addition to nonbiological factors such as climate and temperature [53,58,59]. However, the precise manner and degree by which these factors individually influence honey robbing remain unclear. When the foraging strategy changes, the number of foraging bees in a colony notably increases, and individual worker bees adjust their flight patterns, including frequency, duration, and distance, to meet new foraging needs [4]. During honey robbing, the number of foraging bees in the robbing colony significantly increases. These bees do not directly participate in honey storage and processing but rather focus on transferring the collected honey to the house bees, thereby increasing the overall food-storage efficiency of the robbing colony. Moreover, the guard bees of the robbed colony play a crucial role in defending against the invasion of robber bees [60]. Bees identify their own kind through pheromones. When robber bees invade, the number of guard bees and the rejection rate of non-colony foraging bees significantly increase. The underlying causes of this shift in collective behavior may be revealed through in-depth studies of individual behavior or physiological mechanisms. In one study, sugar-sensitivity tests were conducted on three types of worker bees responsible for collecting nectar, pollen, and water within the colony; the results showed that the bees’ sensitivity to sugar increased in that order [61]. Bees are unlikely to perform honey robbing as a specialized role [45]. We hypothesize that robber bees may be more sensitive to sugar, increasing their attraction to the scent of honey within the robbed colony. Furthermore, studies have shown that, compared with that of normal foraging bees, the lifespan of robber bees is significantly shorter, which may be related to an increase in disease infection rates or accelerated energy metabolism [41,46]. Grume et al. noted that robber bees exhibit high aggression, which is a crucial prerequisite for preying on or stealing food from other colonies [4].

Previous studies have also highlighted the importance of information exchange and coordination mechanisms among robber bees. Hasenjager et al. identified two crucial dance languages involved in honey robbing among bees [62]. One dance is the waggle dance, which is used to recruit bees, and the other dance is a short, rapid vibration dance termed the “stop signal”, signaling the end of recruitment. Both dances are vital in regulating food collection and storage behaviors associated with honey robbing. Specifically, the waggle dance of robber bees appears to guide the house bees during the task of receiving food [63]. During honey robbing, if the number of house bees is not sufficient to quickly receive all the food brought back, the waiting time for robber bees will increase correspondingly. At this point, the robber bees will switch from performing the waggle dance, which originally indicates the direction of the food source, to a trembling dance to attract more house bees to come and receive food [64]. The stop signal is performed by bees through brief vibrations of their bodies, typically lasting approximately 150 ms with a frequency of approximately 380 Hz. If the robbed colony is able to defend itself effectively, obstructing the ability of the robber bees to continue their plunder, the robber bees emit this stop signal upon returning to their own colony. This signal may inform their fellow bees that the current food source is no longer safe and that the robbing needs to stop [65]. In addition, pheromone communication among bee colonies is a crucial form of chemical communication. Studies on honey robbing in stingless bees have highlighted the importance of chemical communication in this process [66]. When stingless bees are exposed to the labial gland pheromone citral, the number of robber bees returning to the robbing colony decreases, whereas the number of guard bees in the robbed colony significantly increases [7]. These findings indicate that citral plays a role in chemical communication during honey robbing among stingless bees, but the specific mechanisms of its action remain to be further elucidated.

To date, studies on honey robbing remain relatively limited, primarily focusing on group behavior at the phenotypic level [2,4], and studies on the specific phenotypes and underlying mechanisms of individual behavioral changes are scarce. Studies on the group or individual behaviors of robber bees face multiple challenges. First, honey robbing is a complex phenomenon that involves interactions between multiple colonies, among individual bees, and between individuals and the environment. To reveal these behavioral phenotypes and the underlying mechanisms involved, a deep understanding of the dynamic changes in honey robbing and reasonable and rigorous experimental protocols for replicable verification are necessary. However, owing to variations in bee species, habitats, experimental conditions, and other factors, standardized experimental methods and operational procedures are lacking. Furthermore, the identification and collection of samples for honey robbing is also a major challenge in research. Honey robbing often exhibits seasonality, which limits the time and location for sample collection. Researchers need to overcome these limitations while also mitigating the interference of environmental factors such as weather and nectar sources to ensure the representativeness and reliability of the samples.

Therefore, to promote in-depth development of research on honey robbing, interdisciplinary collaboration should be strengthened, and comprehensive knowledge and technical methods from multiple fields, such as biology, ecology, and ethology, should be applied. Moreover, more standardized and unified experimental methods and operational procedures must be established to increase the accuracy and reproducibility of the research.

### 6.2. Possible Future Research Directions

A future research trend in the study of honey robbing is to explore the underlying neurobiological mechanisms involved. The decision-making process of honey bees typically relies on their perception of diverse environmental stimuli, and these decisions are encoded and regulated by stable and programmed neural circuits in their brains [67,68]. Specifically, by imaging and recording specific neural circuits in the bee brain [67,69], the activity states of relevant neurons during the decision-making process for foraging can be determined, thereby revealing the neural mechanisms underlying changes in honey robbing. In this research direction, the study of neurotransmitter systems plays a pivotal role. The bee brain contains various neurotransmitters, such as octopamine, serotonin, and dopamine, which play central roles in regulating bee behavioral patterns, emotional responses, and learning abilities [60,70,71]. Exploring the specific roles of these neurotransmitters in the decision-making and behavior-selection processes of bees will help us understand how these neurotransmitters drive honey robbing and provide insights into potential regulatory or intervention methods. For example, we can use specific neurotransmitter agonists or antagonists to modulate the behavior of bees, with the aim of reducing the incidence of honey robbing [72]. Moreover, the application of these chemical modulators should be integrated with environmental management strategies, and targeted drugs or solutions should be developed through functional validation. Another promising research direction is exploring the molecular mechanisms underlying honey robbing. With the aid of advanced biotechnological tools, such as transcriptome sequencing and genome sequencing [73,74], researchers can compare and analyze the differences in gene expression profiles between bees exhibiting normal foraging behavior and those displaying robbing behavior. This approach enables the identification of key molecular targets and metabolic pathways closely associated with changes in honey robbing. Through genetic manipulations such as RNA interference (RNAi) and CRISPR-Cas9 gene editing, researchers can directly regulate the expression of candidate key genes under experimental conditions to validate the functions of these genes [75,76]. These findings will provide a molecular basis for the development of targeted strategies to intervene in the occurrence of honey robbing.

Rapid advancements in smart monitoring technologies provide an opportunity to gradually revolutionize traditional modes of beekeeping and management via these innovative approaches. Remote monitoring systems integrate diverse sensors (including temperature, humidity, and gas monitoring) with efficient data-acquisition devices, enabling real-time capture of environmental information inside and outside the hive, as well as dynamic data on the colony [77,78]. These sensors precisely monitor key parameters such as temperature changes within the colony, honey production, colony size, and activity patterns. When the system detects any abnormal fluctuations, such as a sudden increase in bee activity, an alert mechanism is immediately triggered that notifies the beekeepers in a timely manner, allowing them to take prompt action. The collected data can be properly stored and thoroughly analyzed, leveraging powerful algorithms from machine learning and data mining to elucidate potential patterns and trends related to honey robbing. Specifically, by carefully analyzing the activity patterns of bees at different times, beekeepers can identify close correlations between specific environmental factors (such as weather conditions and temperature levels) and changes in honey robbing. Additionally, through retrospective analysis of past honey robbing events, high-risk periods and specific situations can be effectively identified [78], allowing beekeepers to take necessary preventive measures in advance. Furthermore, drone-assisted monitoring offers unprecedented flexibility and efficiency in terms of colony management. Drones equipped with high-definition cameras and various sensors can patrol above the apiary [78], monitor the condition of the hives and their surrounding environment in real time, and transmit these data instantly to a control center. Using the dynamic monitoring capabilities of drones, beekeepers can swiftly detect any signs of abnormal behavior in colonies, enabling them to take effective prevention and management measures in a timely manner.

## 7. Conclusions

Honey robbing is a manifestation of the complex foraging strategies of bee colonies and poses a significant challenge to the sustainable development of beekeeping. To address this challenge effectively, we must increase our understanding of the behavioral characteristics and underlying mechanisms of individual bees involved in honey robbing. This information will not only help to uncover the survival dynamics and strategies of bee societies but is also crucial for ensuring the healthy development of the beekeeping industry. For example, while foraging activity and aggression are known to increase simultaneously during honey robbing [4,45], the specific cues driving this behavior remain unclear. Furthermore, although studies have shown that the levels of serotonin and dopamine, which are related to aggressive behavior, peak in the bee brain during August and September [60,79], the possible relationship between these biochemical changes and the frequency of honey robbing remains unknown.

In-depth research on honey robbing is urgently needed. By elucidating the mechanisms underlying honey robbing, we can not only gain a more comprehensive understanding of the survival strategies of bee societies but also provide a scientific basis for efficient colony management. This information, in turn, lays a solid theoretical foundation for preserving bee biodiversity and promoting the sustainable development of the beekeeping industry.

## Data Availability

All datasets generated for this study are included in the article.
